# Identifying the Critical Threshold for Long-Term Pediatric Neurological Hospitalizations of the Offspring in Preterm Delivery

**DOI:** 10.3390/jcm10132919

**Published:** 2021-06-29

**Authors:** Shiran Zer, Tamar Wainstock, Eyal Sheiner, Shayna Miodownik, Gali Pariente

**Affiliations:** 1 Department of Obstetrics and Gynecology, Soroka University Medical Center, Ben-Gurion University of the Negev, Beer-Sheva 84101, Israel; prosz1@walla.com (S.Z.); miodowni@post.bgu.ac.il (S.M.); galipa@bgu.ac.il (G.P.); 2 The Department of Public Health, Faculty of Health Sciences, Ben-Gurion University of the Negev, Beer-Sheva 84101, Israel; wainstoc@post.bgu.ac.il

**Keywords:** preterm birth, neurological, pediatric

## Abstract

We opted to investigate whether a critical threshold exists for long-term pediatric neurological morbidity, and cerebral palsy (CP), in preterm delivery, via a population-based cohort analysis. Four study groups were classified according to their gestational age at birth: 24–27.6, 28–31.6, 32–36.6 weeks and term deliveries, evaluating the incidence of long-term hospitalizations of the offspring due to neurological morbidity. Cox proportional hazard models were performed to control for confounders. A Kaplan–Meier survival curve was used to compare the cumulative neurological morbidity incidence for each group. A total of 220,563 deliveries were included: 0.1% (118) occurred at 24–27.6 weeks of gestation, 0.4% (776) occurred at 28–31.6 weeks of gestation, 6% (13,308) occurred at 32–36.6 weeks of gestation and 93% (206,361) at term. In a Cox model, while adjusting for confounders, delivery before 25 weeks had a 3.9-fold risk for long-term neurological morbidity (adjusted HR (hazard ratio) = 3.9, 95% CI (confidence interval) 2.3–6.6; *p* < 0.001). The Kaplan–Meier survival curve demonstrated a linear association between long-term neurological morbidity and decreasing gestational age. In a second Cox model, adjusted for confounders, infants born before 25 weeks of gestation had increased rates of CP (adjusted HR = 62.495% CI 25.6–152.4; *p* < 0.001). In our population, the critical cut-off for long-term neurological complications is delivery before 25 weeks gestation.

## 1. Introduction

Globally, an estimated 15 million infants are born preterm annually [1], and preterm birth (PTB) remains the leading cause of death under the age of 5 years [2]. The World Health Organization (WHO) defines PTB as all births before 37 completed weeks of gestation, with a further sub-division based on gestational age; extremely PTB (delivery at less than 28.0 weeks of gestation), very PTB (delivery between 28.0 and 31.6 weeks of gestation) and moderate to late PTB (delivery between 32.0 and 36.6 weeks of gestation) [1].

Over the past few decades, improvements in modern obstetrics, perinatology, and neonatal care have resulted in an improved survival of the premature infant [3,4,5,6]. Among the improvements is included the widespread application of antenatal glucocorticoid therapy, the introduction of synthetic surfactant, and a tendency towards more aggressive feeding strategies [7]. Thus, premature infants are surviving with major and minor neurodevelopmental morbidities, often resulting in lifelong disability [8]. Neurodevelopmental impairments can include, among other morbidities, cerebral palsy (CP), cognitive dysfunction, and sensory impairments [6,9,10].

As the most common cause of severe physical disability in childhood [11,12], CP defines a group of permanent disorders that has occurred in the developing fetal or infant’s brain, resulting in severe limitation of activity [12,13,14,15]. In CP, motor impairment is often accompanied by disturbances of sensation, perception, cognition, communication and behavior [15]. The overall prevalence of CP is estimated to be 2 per 1000 live births [6,11,16] and its risk is inversely proportional to gestational weight and age at birth, but not necessarily increasing severity [6]. The prevalence of CP expressed by gestational age is reported to be 14.6% in extremely preterm children, 6.2% in very preterm children, and 0.7% in moderate to late preterm compared with 0.11% in term-born children [16,17].

The pathophysiologic mechanisms accounting for the neurodevelopmental disorders in preterm survivors are poorly understood. Perinatal systemic inflammation may sensitize the developing brain to secondary insults and contribute to sustained central nervous system inflammation [18]. Inflammation and related cytokines that can lead to preterm birth may be combined with genetic and epigenetic factors, altering the preterm infant’s brain, making it vulnerable to injury [19,20,21,22].

As neurologic morbidity is one of the devastating outcomes of prematurity, we elected to investigate the association between the grade of prematurity and long-term neurological morbidity of the offspring, in order to set up a critical cut-off at which the long-term neurological morbidity of the offspring would be higher.

## 2. Materials and Methods

We conducted a retrospective population-based cohort study, which included all singleton pregnancies in women who delivered between the years 1991 and 2014. The study was conducted at the Soroka University Medical Center (SUMC). The hospital assists the local population by providing obstetrical and pediatric care being the only tertiary medical center in the Negev district. The Negev occupies 60% of the land of Israel, and SUMC serves the entire population of the region (14% of Israel’s population, approximately 1,190,000) with a birth rate in the southern region showing a positive trend and continues to grow each year [23].

Thus, this study is based on nonselective population data. The Institutional Review Board (in accordance with the Helsinki declaration) approved the study (IRB #0357-19-SOR).

Four groups were evaluated during the study period, based on gestational age, as followed by the WHO (1); extreme PTB: 24 + 0–27 + 6, very PTB: 28 + 0−31 + 6, moderate to late PTB: 32 + 0−36 + 6 weeks of gestation and term deliveries. Neurological morbidity included hospitalizations up to 18 years of age involving a predefined set of ICD-9 (International Classification of Diseases) codes, as recorded in hospital records. Neurological morbidity encompasses movement disorders, developmental disorders, degenerative disorders, psychiatric disorders, and CP (Table S1). Multiple pregnancy, women with lack of prenatal care, women with chromosomal abnormalities or congenital anomalies, and perinatal mortality cases (intrauterine fetal death, intrapartum death, postpartum death) were excluded from the study. All other deliveries were included in the study.

Follow up was terminated if any of the following occurred: the first hospitalization at SUMC for neurological morbidity, any hospitalization which resulted in death, the child reached 18 years of age, or at the end of the study period.

Data were collected from two computerized clinical data sets: the first, perinatal data from the obstetric and gynecologic department at SUMC, including information that was documented by obstetricians directly following delivery. Subsequently, medical secretaries regularly examine the data before it is entered into the database. After evaluating and crossing the hospital documents with prenatal care records, coding is performed. Maximal completeness and accuracy of the databases is fulfilled with these unique measures. The second is a computerized pediatric hospitalization at SUMC (Demog-ICD-9), which includes both demographic data and medical diagnosis during hospitalization. The two databases were cross-linked and merged based on the patients’ ID (mother and child). All diagnoses were classified by the international classification of disease (ICD-9).

Neurological outcomes assessed included hospitalization of the offspring up to 18 years of age due to primary or secondary neurological morbidity, with secondary neurological morbidity defined as hospitalization with neurological morbidity not being the primary diagnosis. These outcomes included at least one diagnosis of the following: movement disorder (including seizures disorders), cerebral palsy (including infantile cerebral palsy unspecified, paraplegia, diplegia of upper limbs, and paralysis unspecified), autism spectrum disorders, eating disorders, psychiatric disease, attention deficit hyperactivity disorder, and developmental disorders. The predefined ICD-9 codes of all diagnoses are detailed in the Supplemental Table (Table S1). In our setting, screening for neurological morbidity is performed at The Institute for Child Development, which provides diagnostic services, treatment, and monitoring for children with developmental disorders from birth to 6 years old. Beyond this age, children diagnosed with developmental disorders are referred to at developmental and neurological clinics at SUMC or in the community. Additionally, the Institute for Child Development has close ties with, and oversees, other ambulatory services. Although diagnoses made at the Institute for Child Development diagnoses are not part of the SUMC hospitalization database, these diagnoses are often recorded as background diagnoses in the database when a child is hospitalized. In Israel, early assessment of developmental disorders routinely takes place at community “Well Baby” centers. If an additional evaluation is required, the children and their families are referred to Child and Family Developmental Centers, where the child is evaluated by a multi-disciplinary care team to ascertain eligibility for service provision. Thus, neurological diagnosis may be captured in the SUMC database when a child is referred for hospitalization by the community clinic. Additionally, the community clinic and SUMC share an online interface, which assists in capturing all diagnoses upon admission.

### Statistical Analysis

Univariable analysis was performed to compare background characteristics between the 4 study groups. The univariable analysis included Chi-square tests for categorical variables and ANOVA tests for continuous variables according to their distribution. The incidence of long-term (up to the age of 18 years) hospitalizations of the offspring due to neurological morbidity was evaluated in the four gestational week age groups. Cumulative incidence morbidity rates were compared using a Kaplan–Meier survival curve, with use of the log-rank test to determine significant differences. Cox proportional hazards models were conducted to compare neurological associated hospitalization risk among offspring born preterm (using dummy variables), divided based on gestational age and term offspring (the reference group), while adjusting for length of follow up. The models adjusted for potential confounders based on the univariable analysis and on the clinical importance of the variables. The cox regression for long-term neurological hospitalizations was adjusted for maternal age, diabetes mellitus, hypertensive disorder, cesarean section and child year birth. The cox regression for cerebral palsy was adjusted for maternal age and child year birth. The mothers in the cohort were entered as clusters to account for dependence between siblings. All analysis was performed using SPSS package 23rd edition (IBM, Armonk, NY, USA) as well as the STATA software 12th edition (StataCorp, College Station, TX, USA).

## 3. Results

During the study period, 220,563 deliveries met the inclusion criteria. Of those, 0.1% (118) occurred at 24–27.6 gestational weeks, 0.4% (776) occurred at 28–31.6 gestational weeks, 6% (13,308) occurred at 32–36.6 gestational weeks and 93% (206,361) were born at term. We did not find any significant obvious trend over the years of the study for distributions of preterm birth.

Table 1 presents the demographic maternal characteristics and immediate perinatal outcomes of the study population according to gestational age at birth. There was no difference in maternal age between the groups. Overall parity increased with increasing gestational age. Rates of smokers were highest among women delivering extreme preterm and decreased with increasing gestational age. As gestational age declined, rates of in-vitro fertilization (IVF) pregnancies and low Apgar scores were higher. Diabetes was more prevalent in women delivered preterm, although not in the group of extremely preterm.

Table 2 reveals the incidence rate of disease-specific neurologic-related hospitalizations of the child in accordance with gestational age at birth. Offspring born prematurely were subjected to significantly more hospitalizations due to movement disorders, cerebral palsy as well as psychiatric disorders compared to term offspring.

**Figure 1:**
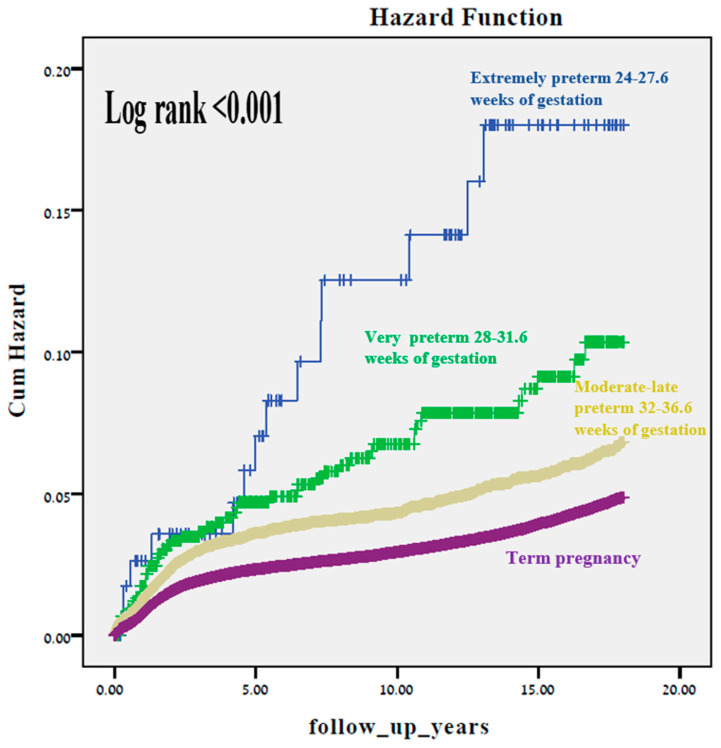
A Kaplan–Meier survival curve exhibiting the cumulative incidence of long-term neurologic-related hospitalizations according to gestational age (Log-Rank test *p* < 0.001).

The Kaplan–Meier survival curve demonstrated a linear association between long-term neurological morbidity and decreasing gestational age (Log-Rank test *p* < 0.001, Figure 1).

**Figure 2:**
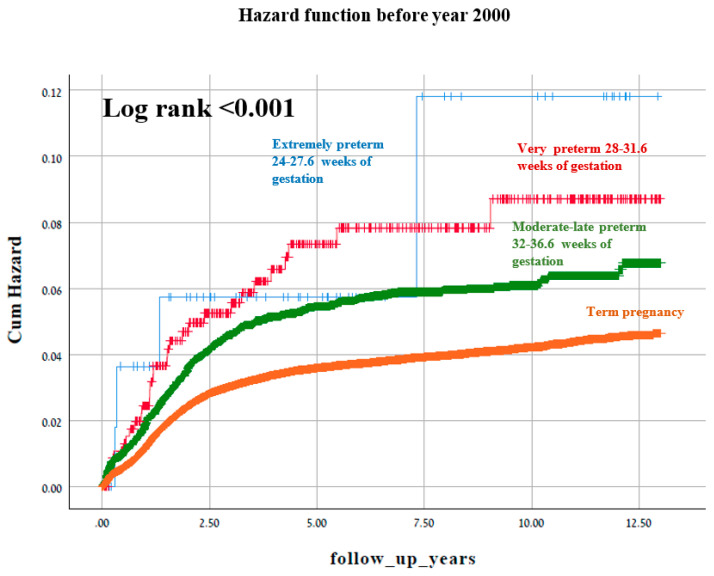
A Kaplan–Meier survival curve demonstrating the cumulative incidence of long-term neurologic-related hospitalizations according to gestational age before year 2000 (Log-Rank test *p* < 0.001).

**Figure 3:**
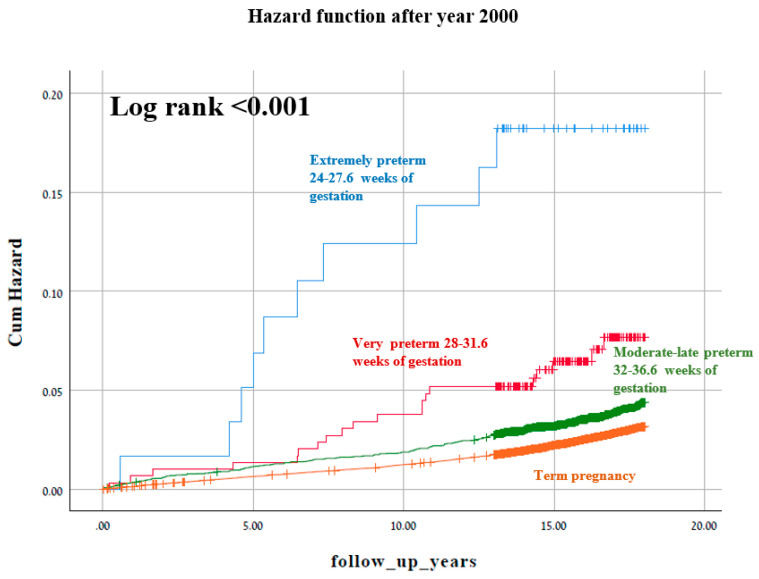
A Kaplan–Meier survival curve demonstrating the cumulative incidence of long-term neurologic-related hospitalizations according to gestational age after year 2000 (Log-Rank test *p* < 0.001).

Figure 2 and Figure 3 represent two Kaplan–Meier curves and each represent the cumulative incidence of long-term neurological hospitalization in two different time periods: 1991–1999 and 2000–2014. No difference in the Log rank test is seen between the two figures.

**Figure 4:**
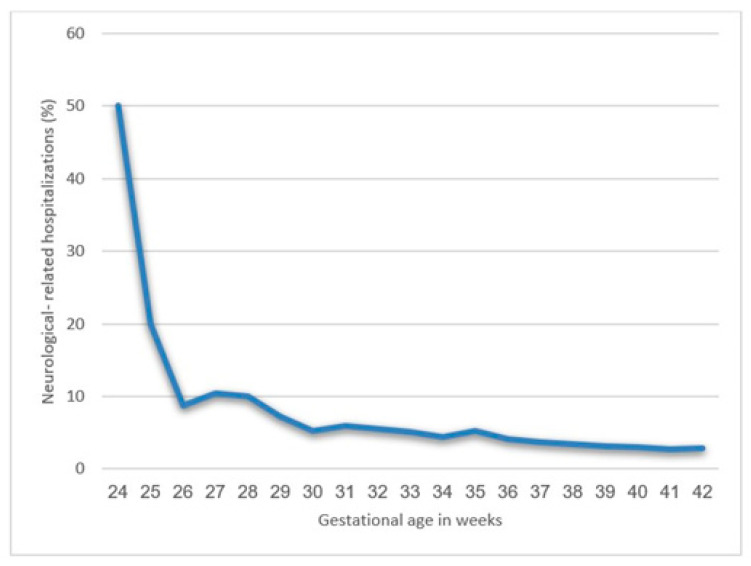
Long-term neurologic-related hospitalizations according to gestational age.

Figure 4 illustrates univariate analysis of total neurological morbidity according to gestational week at delivery. As seen, the gestational week at birth and neurological morbidity are manifested in an inverse relationship. Searching for a specific threshold, the slope for hospitalization rate attenuated beyond 25 weeks of gestation (Figure 2).

Using a Cox model, adjusting for diabetes, hypertensive disorders and cesarean delivery, delivery before 25 weeks had a 3.9-fold risk for long-term neurological morbidity (adjusted HR = 3.9, 95% CI 2.3–6.6; *p* < 0.001, Table 3). In a second Cox model, which adjusted for maternal age, infants born before 25 weeks of gestation had significantly increased rates of CP (adjusted HR = 62.4, 95% CI 25.6–152.4; *p* < 0.001, Table 3). No differences in the medians of follow-up times were demonstrated between the different gestational ages (*p* = 0.179).

**Figure 5:**
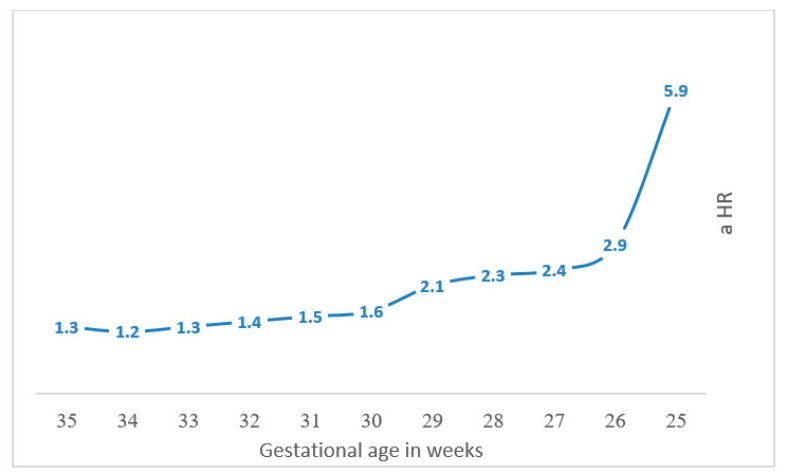
Cox proportional hazard models for long-term neurological morbidity according to gestational age.

Table 4 and Figure 5 exhibit Cox proportional hazard models for long-term neurological morbidity according to gestational age. Twenty five weeks is the critical threshold beyond which long-term neurological morbidity decreased. Another reduction of long-term morbidity is seen between 29 and 30 weeks.

## 4. Discussion

This large retrospective cohort study inspected the long-term incidence of neurologic-related hospitalizations laminated by extremity of prematurity and compared to term offspring. We concluded that decreasing gestational age at birth increased neurological morbidity, as shown in both the Kaplan–Meier survival curve and the Cox regression analysis model. The slope for hospitalization rate according to gestational age attenuated beyond 25 weeks of gestation; therefore, 25 weeks could be determined as the critical threshold below which the risk of neurological morbidity significantly rises, with another threshold effect seen between 29 and 30 weeks, showing a further reduction in long-term neurological morbidity.

These findings are in line with the results of a considerable number of studies in which extremely preterm infants exhibited greater neurodevelopmental impairment than their older gestational age peers [24,25,26]. Pascal et al., systematic review and meta-analysis demonstrated that overall CP prevalence, along with motor and cognitive delays, were higher in extremely low birthweight infants than in very low birth infants. This variability was only statically significant for CP (*p* < 0.001) and motor delays (*p* = 0.012) [24].

Previous studies from our cohort have investigated the possibility of a critical threshold regarding extremity of prematurity and long-term morbidity of the offspring. A large retrospective population-based study by Davidesko et al. found that children born after 32 weeks gestation were at decreased risk of long-term infectious morbidity [27]. Additionally, Ohana et al. demonstrated a relationship between the degree of prematurity and long-term gastrointestinal morbidity of the offspring, with a critical cut-off at 25 weeks gestation [28].

Studies have indicated that the prevalence of CP among extremely preterm children ranges from 16 to 21% [29]. The EPIPAGE Cohort (étude épidémiologique sur les petits âges gestationnels) study evaluated infants born between 22 and 32 weeks’ gestation in nine regions of France in 1997. This study found that at 2 years of age, the prevalence of CP was 20% in those born at 24–26 weeks gestation compared with 4% in those born at 32 weeks [30].

Our study has also demonstrated an independent association between gestational age at birth and the risk for CP. This association may reflect that premature exposure to the extra-uterine environment, including gravity and sensory experiences, can alter musculoskeletal and nervous system development, thereby shifting the trajectory of motor development for otherwise healthy children [26]. Higher rates of CP in the preterm population study groups could be explained by reductions in the rates of post-term births and/or improvements in the accuracy of pregnancy dating over the last decades could have influenced the outcomes of the study.

The human brain develops and matures during the fetal period and is controlled by a set of complex relations among various signaling receptors, genetic/epigenetic factors as well as environmental influences. During the gestational period, several stages of development have been marked: primary neurulation (weeks 3–4), prosencephalic development (months 2–3), neuronal proliferation (months 3–4), neuronal migration (months 3–5), neuronal organization (5 months postnatal period), and myelination. The process of myelination of the human brain spreads from the fetal second trimester and continues postnatally into adulthood, with the quickest growth striking in the immediate neonatal period [31]. The preterm brain is particularly vulnerable because it is exposed to the extra-uterine environment during critical periods of brain development, and is thus at risk of alterations to the “normal” trajectory of brain development [13].

The etiology of neurodevelopmental morbidity, including CP, remains unclear but is thought to be multifactorial. There is increasing evidence that intrauterine or early postnatal inflammation may play a role in the development of CP [32]. Bountiful literature has identified in utero microbial infection and/or inflammation as a strong risk factor for PTB, particularly early spontaneous PTB [33]. The theory is that maternal infection and/or inflammation, occurring during critical periods of fetal development, could alter brain structure and function in a time-sensitive manner, as evidenced by different types of studies. Specifically, in humans, both bacterial and viral infections are associated with abnormal brain structure in affected individuals, while bacterial infections during gestation have been weakly associated with abnormal psychological and cognitive development in their offspring. Data gathered from animal models and retrospective human data and findings advocate potential causative mechanisms for the correlation between injury to the fetal brain and infection; specifically, the inflammatory cascade, caused by infection, is characterized by elevations in various cytokines, such as IL-6, eventually ensuing altered brain structure and function [34].

Amongst the strengths of our study is the population-based nature of its data, and the fact that our hospital serves as only a tertiary medical center of the entire southern region. As a consequence, the majority of the patients are provided with health care services in our facility. The robustness of our conclusions relies on the population-based nature of the cohort, without a selection bias. In our study, vast inclusion criteria and limited exclusion criteria yielded a representative study population. Nevertheless, various limitations in our results should be addressed. The retrospective nature of the data, which are based on a database registry, which has intrinsic limitations related to the type of retrieved information as well as the possibility of misclassification of the outcome (neurological morbidity), provide a major limitation. Most of the neurological conditions evaluated in this study are routinely diagnosed and treated in an ambulatory setting. Thus, our hospital-based database may not reflect the true population morbidity, and some cases will be missed unless the child is hospitalized for any reason. Furthermore, in the case of several of the neurological outcomes, diagnosis is performed via specialized screening. It is therefore possible that there are patients who have the disorder but have not ever been screened. These factors put our study at risk of selection bias. Nevertheless, because some of the conditions included in the study are associated with significant health burdens and comorbidities, sufferers are likely to be hospitalized at one time or another.

Deviation of the results toward the null hypothesis could be caused by this bias. Merely grave neurological morbidities, leading to hospitalizations, were identified, and classified as such. Thus, our conclusions must be restricted to such cases. Even though the southern region is usually influenced by positive immigration, the possibility of immigration of children born outside the hospital coverage zone remains. Another important limitation relates to the study follow-up of the offspring, until the child reaches 18 years old. Various morbidities could arise only at an older age. This deserves to be investigated in future studies. In addition, data regarding interventions to improve perinatal outcomes of preterm infants, including GBS prophylaxis, use of corticosteroids for lung maturation, as well as neuroprotection with magnesium sulfate, were not available for further statistical analysis.

## 5. Conclusions

In conclusion, very preterm offspring have the highest risk for CP and long-term neurological morbidity. A crucial threshold of 25 weeks gestation was prominent in our study, below which the jeopardy of long-term neurological morbidity of the offspring increased significantly. It is also emphasized that each additional week of gestation further decreased the risk of long-term neurological morbidity, stressing the necessity to consider to properly schedule medically indicated preterm delivery and even early-term delivery in respect of the long-term health of the offspring. Clinical practice should attempt prevention of preterm delivery where possible and attempt to optimize the timing of medically indicted induced delivery as close to full term as medically possible without surpassing an unacceptable increase in the risk to both mother and fetus. Increased surveillance during childhood for signs of neurological impairment could contribute to offspring born prematurely, which is unique for those who born prior to 25 weeks of completed gestation. The mechanisms by which in utero events impact brain injury resulting in subsequent adverse neurodevelopment are extraordinarily complex. Understanding the pathways that lead to observed associations is a challenge that will require immense ongoing scientific rigor.

## Figures and Tables

**Table 1:** Maternal characteristics and immediate perinatal outcomes of the study population according to gestational age at birth.

Characteristics	Extreme PTB (*n* = 118) (%)	Very PTB (*n* = 776) (%)	Moderate-Late PTB (*n* = 13,308) (%)	Term Birth (*n* = 206,361) (%)	*p*-Value
Maternal Age, years (mean ± SD)	28.47 ± 6.34	28.36 ± 6.37	28.31 ± 6.24	28.24 ± 5.76	0.482
Gravidity					
1	29.7	24.5	23.3	20.2	<0.001
2–4	46.6	44.8	44.5	48.7	
5+	23.7	30.7	32.1	31.2	
Parity					
1	38.1	31.4	29.0	24.2	
2–4	48.3	47.7	47.7	52.0	<0.001
5+	13.6	20.9	23.3	23.8	
Smokers	4.2	1	1.5	1	<0.001
Fertility treatments					<0.001
In vitro fertilization	3.4	2.6	2.3	1.1	
Ovulation induction	1.7	1.8	1.1	0.8	
Hypertensive disorders *	8.5	19.1	12.9	4.7	<0.001
Maternal diabetes **	0.0	6.2	8.1	5.2	<0.001
Induction of labor	14.3	4	27.3	31.8	<0.001
Type of birth					<0.001
Vaginal delivery	48.3	46.5	67.3	83.9	
Assisted vaginal delivery	0	0.6	1.7	3.3	
Cesarean delivery	51.7	52.8	31.0	12.7	
Small for gestational age (SGA)	3.4	1.8	3.6	4.4	<0.001
Apgar score 1 min < 7	49.2	28.1	10.2	4.1	<0.001
Apgar score 5 min < 7	14.4	6.2	2.5	1.4	<0.001

* Hypertensive disorders include chronic hypertension, gestational hypertension preeclampsia and eclampsia. ** Maternal diabetes includes pre-gestational diabetes and gestational diabetes. PTB, preterm birth; SD, standard deviation.

**Table 2:** Incidence rate of disease-specific hospitalizations according to gestational age at birth.

Neurological Morbidity of the Offspring	Extreme PTB (*n* = 118) (%)	Very PTB (*n* = 776) (%)	Moderate-Late PTB (*n* = 13,308) (%)	Term Birth *n*= (206,361) (%)	*p*-Value
Movement disorders	4.2	3.5	2.7	1.8	<0.001
Cerebral palsy	4.2	0.9	0.2	0.1	<0.001
Psychiatric disorders	2.5	0.9	0.7	0.5	<0.001
Developmental disorders	0.8	0.3	0.2	0.1	<0.001
Degenerative disorders	0	0.6	0.1	0.1	<0.001

**Table 3:** Cox multivariable analyses for long-term neurologic-related hospitalizations and for CP according to gestational age.

	Total Neurologic Related Hospitalizations				Cerebral Palsy	
Gestational Age	aHR *	95% CI	*p*-Value	aHR **	95% CI	*p*-Value
Term delivery (reference) >37 gestational weeks	1	-	-	1	-	-
Moderate to late preterm	1.3	1.2–1.5	<0.001	2.5	1.6–3.9	<0.001
Very preterm	1.9	1.4–2.5	<0.001	13.4	6.2–28.7	<0.001
Extremely preterm	3.9	2.3–6.6	<0.001	62.4	25.6–152.4	<0.001

* Adjusted for maternal age, diabetes mellitus, hypertensive disorders and cesarean section and childbirth year. ** Adjusted for maternal age and childbirth year. aHR, adjusted hazard ratio, CI, confidence interval.

**Table 4:** Cox proportional hazards models for long-term neurological morbidity according to gestational age.

		Total Neurologic Related Hospitalizations		
	Gestational Age	aHR *	95% CI	*p*-Value
Model 1	PTB 25 gestational week versus other later weeks of PTB	5.9	2.2–15.9	<0.001
Model 2	PTB 26 gestational week versus other later weeks of PTB	2.9	1.4–6.2	<0.004
Model 3	PTB 27 gestational week versus other later weeks of PTB	2.4	1.4–4.2	<0.001
Model 4	PTB 28 gestational week versus other later weeks of PTB	2.3	1.5–3.5	<0.001
Model 5	PTB 29 gestational week versus other later weeks of PTB	2.1	1.4–3.0	<0.001
Model 6	PTB 30 gestational week versus other later weeks of PTB	1.6	1.2–2.2	<0.002
Model 7	PTB 31 gestational week versus other later weeks of PTB	1.5	1.1–1.9	<0.002
Model 8	PTB 32 gestational week versus other later weeks of PTB	1.4	1.1–1.7	<0.003
Model 9	PTB 33 gestational week versus other later weeks of PTB	1.3	1.1–1.6	<0.003
Model 10	PTB 34 gestational week versus other later weeks of PTB	1.2	1.0–1.4	<0.03
Model 11	PTB 35 gestational week versus other later weeks of PTB	1.3	1.1–1.5	<0.001

* Adjusted for maternal age, diabetes mellitus, hypertensive disorders and cesarean section.

## Data Availability

Due to IRB terms supporting data not provided.

## Supplementary Materials

The following are available online at https://www.mdpi.com/article/10.3390/jcm10132919/s1, Table S1: ICD-9 codes of neurological disorders considered in the study.
